# Monkey’s Social Roles Predict Their Affective Reactivity

**DOI:** 10.1007/s42761-021-00048-8

**Published:** 2021-07-27

**Authors:** Eliza Bliss-Moreau, Anthony C. Santistevan, Brianne Beisner, Gilda Moadab, Jessica Vandeleest, Brenda McCowan

**Affiliations:** 1 Department of Psychology, University of California Davis, Davis, CA 95616 USA; 2 California National Primate Research Center, University of California Davis, Davis, CA 95616 USA; 3 Department of Population Health and Reproduction, School of Veterinary Medicine, University of California Davis, Davis, CA USA

**Keywords:** Social networksSocial network analysisRankNonhuman primates*Macaca mulatta*Affective reactivity

## Abstract

Accumulating evidence demonstrates that the number of social connections an individual has predicts health and wellbeing outcomes in people and nonhuman animals. In this report, we investigate the relationship between features of an individuals’ role within his social network and affective reactivity to ostensibly threatening stimuli, using a highly translatable animal model — rhesus monkeys. Features of the social network were quantified via observations of one large (0.5 acre) cage that included 83 adult monkeys. The affective reactivity profiles of twenty adult male monkeys were subsequently evaluated in two classic laboratory-based tasks of negative affective reactivity (human intruder and object responsiveness). Rhesus monkeys who had greater social status, characterized by age, higher rank, more close social partners, and who themselves have more close social partners, and who played a more central social role in their affiliative network were less reactive on both tasks. While links between social roles and social status and psychological processes have been demonstrated, these data provide new insights about the link between social status and affective processes in a tractable animal model of human health and disease.

While it has long been recognized that features of people’s social networks and their position in those social networks are related to health and wellbeing (House et al., 1988), recent research demonstrates that aspects of a person’s psychology, and, in particular, features related to socioaffective processes, impact the structure of their social network and the social role they play in their network. For example, there is evidence that people’s self-reported empathic capacity is related to the size of their social networks (Kardos et al., 2017) and the importance of their social role in networks related to interpersonal trust (Morelli et al., 2017). Further, people’s social relationships promote or buffer them against negative psychological experiences and outcomes, and people with higher social status tend to have better long-term psychological outcomes (e.g., Adler et al., 2000; Cohen & Hoberman, 1983). Together, these findings point to an interesting and important, potentially cyclical relationship between individual-level psychological function and group-level social network characteristics — certain people appear to have complex social roles in large social networks and the social features characteristic of such individuals may feedback to improve their wellbeing. Understanding the causal relationships between features of people and their social networks is critical for generating social interventions aimed at improving people’s lives and understanding how individual-level psychological features come to be. Yet, studies of humans leave open many questions about these relationships, including those about their developmental (proximate) and evolutionary (ultimate) origins as well as the importance of features of social networks, which are not easily measured (e.g., social reach in face-to-face relationships, transitivity in relationships).

Here, we adopt a highly translatable animal model for studying social and affective processing, rhesus monkeys (*Macaca mulatta*), in order to test the hypothesis that individual level psychological features relate to a monkey’s role in his social network. Rhesus monkeys are one of the best animal models for the study of social and affective processing in humans. They live in large, complex multi-male multi-female social groups and maintain social bonds via repertoires of nuanced social and affective behaviors. They share a number of homologies with humans related to social and affective processes (e.g., context dependent social signaling; Beisner & McCowan, 2014), encoding of valence via the autonomic nervous system (Bliss-Moreau et al., 2013 etc.), and specific homologies related to how social relationships buffer against negative or poor psychological outcomes (for an example, see Hennessy et al., 2017; for a review on monkey models, see Phillips et al., 2014). These behaviors are generated by complex brains that share many homologies with humans, including features like highly developed prefrontal cortex, which rodents lack (Laubach et al., 2018; Wise, 2008).

We adopt social network metrics that are both directly translatable to and measurable in humans (e.g., how many “friends” a monkey has) as well as those that are directly translatable but not easily measured or modeled in humans either because all possible social relationships are not known or because members of human social groups are in flux (e.g., social reach, transitivity, rank among all possible social partners in the “friends” network). Here, we use grooming relationships as a proxy for close social relationships that may be similar to human friendships. While grooming provides specific health advantages (e.g., reducing parasite load; Akinyi et al., 2013), one of its primary functions in nonhuman primates is forming and maintaining social bonds. Grooming is directed (e.g., “I groom you” is different from “you groom me”) and grooming relationships are often reciprocated between partners (e.g., “I groom you and you groom me”; Shino & Aureli, 2008). Animals often groom up the status hierarchy in order to secure support during agonistic interactions (Schino, 2007) and individual-level grooming relationships are important for group-level social cohesion (Dunbar, 1991; Lehmann et al., 2007). Given that the frequency of other affiliative behaviors like play is relatively low in adult rhesus monkeys, we reasoned that grooming relationships would be the best proxy for the sorts of social network metrics used in humans that ask people to report on their number of friends.

Direct behavioral observations of monkeys interacting within a closed or contained system have the added benefit of creating an objective record of all possible interactions and interaction partners, without reliance on self-reports to construct networks that represent social interactions (e.g., the Social Network Index; Cohen et al., 1997). Like humans, rhesus monkeys’ social relationships and roles in their social networks have clear implications for their health and wellbeing (for a review, see McCowan et al., 2016) and social network features that are not typically measured in humans seem to play a particularly important role in heath in rhesus monkeys, including how certain animals are of their status (Vandeleest et al., 2016) and the number of indirect social connections (or “friends of friends”) they have (for a review, see Brent, 2015) and the importance or centrality of their social role (how many individuals and groups they connect) (Duboscq et al., 2016).

Here, we model the social network of a closed, large group of rhesus monkeys to test the hypothesis that features of their social roles, including how many direct and indirect positive social relationships they have (e.g., prosocial as measured by grooming, rather than aggressive or antagonistic), the importance of their social role (betweenness centrality), rank, and rank certainty are related to individual differences in affective reactivity — the robustness of affective response to potential threat — measured in the laboratory using two well established protocols: Human Intruder Testing and Object Responsiveness Testing (Bliss-Moreau et al., 2010; Bliss-Moreau et al., 2011; Gottlieb & Capitanio, 2013; Kalin & Shelton, 1998; Mason et al., 2006). Both of these tasks are widely used and are thought to establish a sort of ground truth about an individual’s capacity for affective reactivity in negative situations (for a discussion see Bliss-Moreau, et al., submitted). We were specifically interested in responsivity to threat given the literature in humans, which has documented that social relationships buffer against negative psychological outcomes.

## Methods

All procedures were approved by the University of California Davis Institutional Animal Care and Use Committee and carried out at the California National Primate Research Center (CNPRC).

### Subjects

Subjects were 20 adult male rhesus monkeys (*Macaca mulatta*) living in one of the 0.5 acres outdoor corrals at the CNPRC. At the beginning of laboratory-based testing, there were 83 adult (defined as greater than 3 years of age) monkeys living in the corral (*N*=58 females and *N*=25 males). Three adult males were enrolled in other investigators’ studies and so were not included in this study; one of the males was hospitalized during the study and so he and his assigned social partner were not included. All other males (*N*=20) were enrolled. When the affective reactivity testing began, the age range of subjects was 3.81 to 14.98 (mean = 7.42, *SD* = 4.22). All animals in the cage were observed in order to compute the social network statistics, but only the 20 subjects completed affective reactivity testing detailed below. In the outdoor corral, animals were fed monkey chow twice daily, provided with fruits and vegetables multiple times a week, and had unlimited access to water.

### Social Network Observations

Prior to the beginning of laboratory-based testing, three observers collected data for 6 h on each of two observation days per week for a total of 8 weeks using a scan sampling technique (1 scan/20 min; Altmann, 1974). Data on grooming relationships was used in the present analysis, including directionality (who groomed whom). Krippendorf’s alpha was used to compute reliabilities and all observers had agreement about 0.85 for behaviors and 0.95 for individual identifications; reliabilities were reassessed at least once per year. This generated a total of 280 scans from which the networks were built. Edge lists with directional grooming data were subjected to social network analyses in UCINet (Borgatti et al., 2002) from which in-degree (number of animals grooming subject), out-degree (number of animals groomed by subject), in-degree two step (number of animals grooming animals grooming subject), out-degree two step (number of animals groomed by animals groomed by subject), and betweenness centrality (how central or important the individuals’ social role is in the network) were extracted.

Aggressive interactions were recorded with concurrent event sampling to determine ordinal dominance rank and dominance probability using the “Perc” package in R (Fujii et al., 2019; Fushing et al., 2011). Interactions were recorded from, and ordinal ranking included, all animals in the cage (not just the subjects). For ordinal rank, low values are higher rank and higher values are lower rank. Dominance probability is an index of transitivity of dominance-related behaviors that includes indirect relationships. If it is high (close to 1), then dominance signaling is consistent across individuals such that there is agreement among animals with regards to the dominance status of the subject. If it is low (close to 0.5), then dominance signaling is inconsistent across individuals such that there is not agreement among animals with regards to the dominance status of the subject.

### Affective Reactivity Testing

Laboratory-based testing occurred 48 days after the conclusion of the social network observations, detailed above. Subjects were relocated into an indoor testing room into standard primate caging (61 cm W × 66 cm D × 81 cm H) in pairs; animals were individually housed but could see the other monkey via large mirrors placed in the room. Pairs were selected such that no pair had been observed to engage in aggression. Animals were relocated to the indoor testing room by CNPRC technical staff at approximately 1400 hours on day 1 and returned to the corral at approximately 1500 hours on day 2, thus spending approximately 25 h indoors. Indoor rooms were maintained at 25 °C and on a 12/12 light/dark cycle with lights on at 0600 and off at 1800. Prior to lights out at 1800, monkeys were fed standard rations of monkey chow, given 1 h to eat, and then all remaining chow was removed from their cage. Animals had ad lib access to water.

Monkeys completed two affective reactivity tests — the Human Intruder Test on day 1 and the Object Responsiveness Test on day 2.

#### Human Intruder Testing (HIT)

HIT occurred ~2 h 15 min after the monkeys were relocated indoors. Monkeys were moved one at a time from their housing room to an adjacent test room and placed in a standard primate cage (61 cm W × 66 cm D × 81 cm H) that did not have perch bars or enrichment items (e.g., mirrors, toys). Each monkey was tested individually while his social partner remained in the housing room. They were given 1 min to acclimate to the room, before a novel experimenter — the “intruder” — entered the room. The intruder stood in front of the monkey for 4 1-min successive trials during which she varied her body position. During trial 1, she stood 3 feet from the front of the cage with her shoulder directed at the cage (so that the profile of her face was visible to the monkey; *Profile Far*). During trial 2, she stood in the same orientation but 1 foot from the cage (*Profile Near*). During trial 3, she stood 3 feet from the cage and faced the monkey, making eye contact with him for the duration of the trial (*Stare Far*). During trial 4, she stood 1 foot from the cage and faced the monkey, making eye contact with him for the duration of the trial (*Stare Near*). Trials were recorded with a Logitech webcam mounted on an extended tripod and recorded from a laptop computer in the anteroom just outside of the testing room. The intruder did not interact with the monkeys prior to, or after, HIT.

HIT data were scored according to standard scoring procedures (Bliss-Moreau & Baxter, 2018) which recorded the location of the monkey (i.e., front of cage, back of cage, on floor of cage, off floor of cage) as well as specific behaviors that constituted the affective reactivity score: facial displays (i.e., bared teeth, lipsmack, threat), vocalizations (i.e., bark, grunt, scream), body postures and physical displays (i.e., cage shake, crooktail, freeze), and non-specific behaviors thought to be indicative of negative affect in affect inducing contexts (i.e., scratch, tooth grind, yawn) and stereotypies. Each 1-min trial was scored in 6 10-s blocks using 0/1 scoring. If a behavior was present during the 10-s block, it was scored as a 1. If a behavior began during the 10-s block but continued into the next 10-s block, a score of 1 was placed in each block. A reactivity score was then computed by summing the 0/1 values across each of the 10-s blocks per trial.

#### Object Responsiveness Testing (ORT)

ORT occurred at approximately 1315 hours on day 2 following a similar protocol to those previously used by our lab (Bliss-Moreau et al., 2011). Testing occurred in the room and cage in which the monkey had spent the night. An opaque divider (~7′ high × 4′ wide) was positioned between the two cages, at a 90° angle into the room, so that the monkeys could not see each other or the testing apparatus when the other animal was testing. A wheeled wired bookshelf was configured such that the top shelf was at the same vertical height as the bottom of the monkey’s cage and was wheeled in front of the cage for testing. Objects were placed on the shelf in front of the monkey within reach. We selected objects based on data from another affective reactivity experiment that measured objects’ potency for generating affective behavioral responses in adult male rhesus monkeys (Bliss-Moreau et al., submitted). Objects were either potentially threatening animal replicas all ~8″ to 12″ length (a plastic dinosaur, a plastic frilled lizard, a plastic horned lizard) or control objects that were ostensibly neutral (a green star ice cube tray, a basket, a small plastic child’s badminton racket) — objects were roughly matched for size. It is important to note that monkeys living outdoors likely have had experience with snakes, spiders, and lizards, including those with the potential to cause significant physical harm to them (e.g., rattlesnakes and black widow spiders) as there are many species of snakes, lizards, and spiders native to our area. Each trial lasted 1 min with approximately a 15-s inter trial interval (ITI was the time to remove the object, place it into a storage box on the other side of the room, and then place the new object on the platform). Objects were presented in one of two orders, counterbalanced across subjects; trial types were intermixed: control-animal-control, etc. or animal-control-animal, etc. (that is, there was never a case where two control objects or two reptile objects were presented one after the other).

Behaviors were recorded with the Logitech webcam on a laptop computer in the anteroom immediately adjacent to the room where the animals were located. Data were scored with our standard ethogram (Bliss-Moreau et al., 2011) using Noldus Observer 5.0. The frequency of behaviors related to affective reactivity (e.g., communicative signals, displays) was summed for both control trials and animal trials to compute the measure of affective reactivity. Those behaviors included facial displays (i.e., bared teeth, lipsmack, threat), body postures and physical displays (i.e., cage shake, crooktail, freeze), vocalizations (i.e., bark, coo, grunt, scream), non-specific behaviors thought to be indicative of negative affect in affect inducing contexts (i.e., scratch, self-sex, self-groom, self-shake, tooth grind, yawn), and stereotypies (including eye covering).

#### Data and Analysis

Data analyses were carried out in SPSS 25.0 (IBM Corp). Metrics of interest from the social network analyses of the grooming network data were highly correlated (see Table 1) across animals. As a result, we subjected the social network data (including dominance probability) to a principal components analysis and then used the resulting component scores in analyses related to behavioral affective reactivity. Because the overall rates of behavior in the two threat processing tasks were relatively low, we created a total score for affective reactivity over both tasks and used that as the affective reactivity variable.

## Results

### Social Network Characteristics

Individual level features of the social network were highly correlated with each other and with age and ordinal rank, although not with dominance probability (Table 1).

**Table 1:** Summary statistics and relationships between independent variables

	Mean (*sd*)	Correlations							Rotated component loadings						
		Age	Rank	In-degree	In-degree (2 step)	Betweenness	Out-degree	Out-degree (2 step)	Solution 1		Solution 2			Solution 3	
									C1	C2	C1	C2	C3	C1	C2
Age (years)	7.65 (4.17)								**.93**	−.23	**.87**	.29	.31	**.90**	.29
Rank	47.20 (32.50)	−.93***							−**.93**	.12	−**.84**	−.35	−.21	−**.86**	−.35
In-degree	8.75 (5.97)	.85***	−.84***						**.91**	−.14	**.96**	.16	.04	**.95**	.19
In-degree (2 step)	42.55 (23.16)	.85***	−.85***	.91***					**.92**	.02	**.91**	.30	−.05	**.89**	.32
Betweenness	87.30 (73.77)	.79***	−.73***	.89***	.79***				**.89**	−.02	**.83**	.34	.07	**.83**	.36
Out-degree	7.90 (2.83)	.57**	−.60**	.43	.53*	.56*			**.72**	**.43**	.30	**.91**	.13	.30	**.91**
Out-degree (2 step)	45.35 (12.89)	.48*	−.55*	.48*	.58**	.58**	.84***		**.70**	**.60**	.33	**.90**	−.12	.29	**.91**
Dominance probability	.87 (.04)	.38	−.28	.18	.08	.19	.14	−.05	.27	−**.75**	.13	0.0	**.98**	-	-

* *p* < 0.05; ** *p* < 0.01; *** *p* < 0.001. Rotated component loadings reflect the varimax rotated solutions, for the 2 component and 3 component solutions. C1 – C3 = component 1 – component 3. Bolded component loadings indicate loading > 0.4. Principal component scores for components 1 and 2 in solution 3 were used in subsequent regression analyses. Note that low rank values indicate high rank (e.g., animal ranked 1 is the highest ranking in the group) which accounts for the negative component loading of rank onto C1

Given the correlations between our predictors of interest, we opted to carry out a principal components analysis on the data with varimax rotation and Kaiser normalization. The initial analysis (Table 1, Solution 1) produced two components, with all predictors except dominance probability loading strongly onto component 1 and dominance probability, out-degree, and out-degree (two step) loading strongly onto component 2. The first component explained 65.89% of the variance in the unrotated solution (eigen value = 5.27) and 65.87% of the variance in the rotated solution (rotated sum of squares loading = 5.27). The second component (dominance probability alone) explained 14.78% of the variance in the unrotated solution (eigen value = 1.18) and 14.78% of the variance in the rotated solution (rotated sum of squares loading = 1.18). The components together explained 80.67% of the variance.

Given the strong cross-loading of out-degree and out-degree (two step) on rotated components 1 and 2, we additionally investigated the rotated three component solution (Table 1, Solution 2). The variables which loaded onto the first component of the three-component solution included age, ordinal rank, number of grooming partners who groom the subject, number of grooming partners who groom the grooming partners of the subject, and betweenness, explaining 65.89% of the variance in the unrotated solution (eigen value = 5.27) and 51.54% of the variance in the rotated solution (rotated sum of squares loading = 4.12). Number of grooming partners whom the subject groomed and the number of grooming partners who the grooming partners of the subject groomed loaded onto the second component and explained an additional 14.78% of the variance in the unrotated solution (eigen value = 1.18) and 25.84% of the variance in the rotated solution (rotated sum of squares loading = 2.07). The third component was dominance probability alone which explained 10.94% of the variance in the unrotated solution (eigen value = 0.88) and 14.23% of the variance in the rotated solution (rotated sum of squares loading = 1.14). Dominance probability consisting of its own component is also evidenced by observing the correlation structure between the variables, which shows that dominance probability was not significantly correlated with any of the other variables of interest. The total variance explained by all three components in the rotated solution was 91.61%.

In order to not have a component constituted by a single variable, and because dominance probability was not significantly correlated with the other variables, we re-ran the principal components analysis dropping dominance probability from the PCA altogether (Table 1, Solution 3). In this final two-component solution, age, ordinal rank, number of grooming partners who groom the subject, number of grooming partners who groom the grooming partners of the subject, and betweenness loaded onto the first component which explained 74.56% of the variance in the unrotated solution (eigen value = 5.22) and 58.53% of the variance in the rotated solution (rotated sum of squares loading = 4.10). We conceptualize this component as representing “social status.” Number of grooming partners whom the subject groomed and the number of grooming partners who the grooming partners of the subject groomed loaded onto the second component and explained an additional 14.54% of the variance in the unrotated solution (eigen value = 1.02) and 30.56% of the variance in the rotated solution (rotated sum of squares loading = 2.14). We conceptualize this component as representing “social motivation.” The components together explained 89.09% of the variance in the rotated solution. We used the regression method to compute individual level component scores from this final solution which were used in subsequent analyses.

### Affective Reactivity

#### HIT

**Fig. 1:**
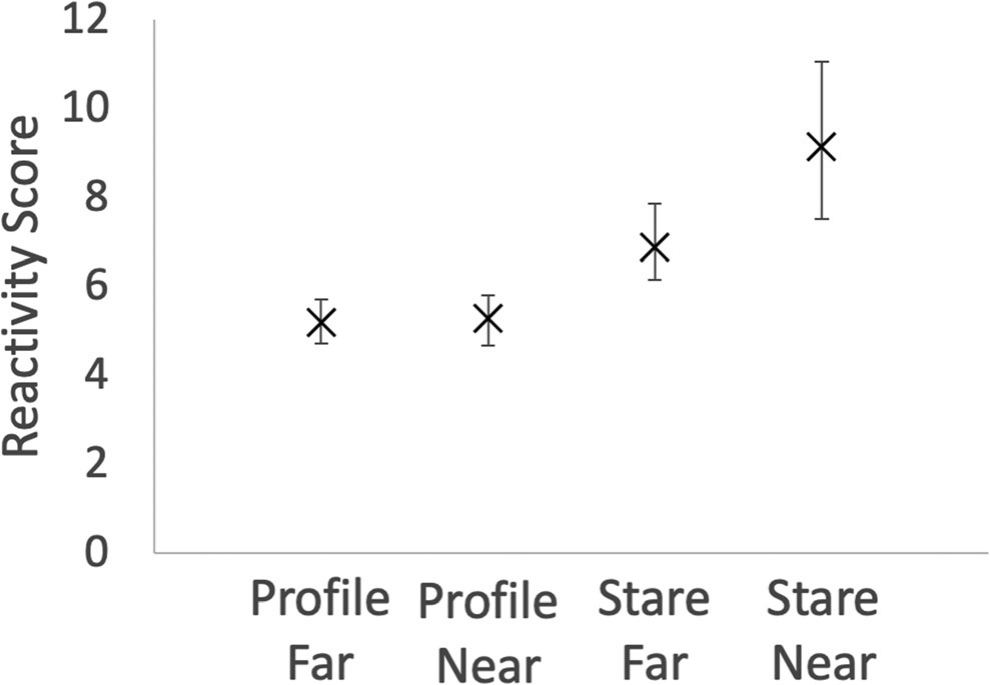
Affective reactivity during HIT. X represents means; error bars are 95% confidence intervals. Trial types are represented on the x-axis, with two body positions (Profile and Stare) and two distances (Far and Near)

Animals behaved as expected during HIT insofar as their reactivity varied across conditions, Friedman’s two-way analysis of variance by ranks test statistic=31.38, *p*<0.0001. As in previous experiments, animals were significantly more reactive to the Stare than Profile conditions, Wilcoxon signed-ranks test *Z*= 3.47, *p*<0.001. They responded essentially identically to the two profile conditions Wilcoxon signed-ranks test *Z*=0.43, *p*=0.67, but were more reactive during the Stare Near than Stare Far condition, Wilcoxon signed-ranks test *Z*=2.52, *p*<0.012 (Fig. 1).

#### ORT

Animals’ reactivity to reptile objects (*M*=3.80, *SD*=4.43) was greater than the reactivity to control objects (*M*=1.85, *SD*=3.05) but not significantly so, Wilcoxon signed-ranks test *Z*=1.50, *p*=0.132.

#### Relationship Between HIT and ORT

Affective reactivity across all HIT and ORT trials were correlated as expected, *r*=0.56, *p* =0.01.

### Relationship Between Social Role and Affective Reactivity

**Fig. 2:**
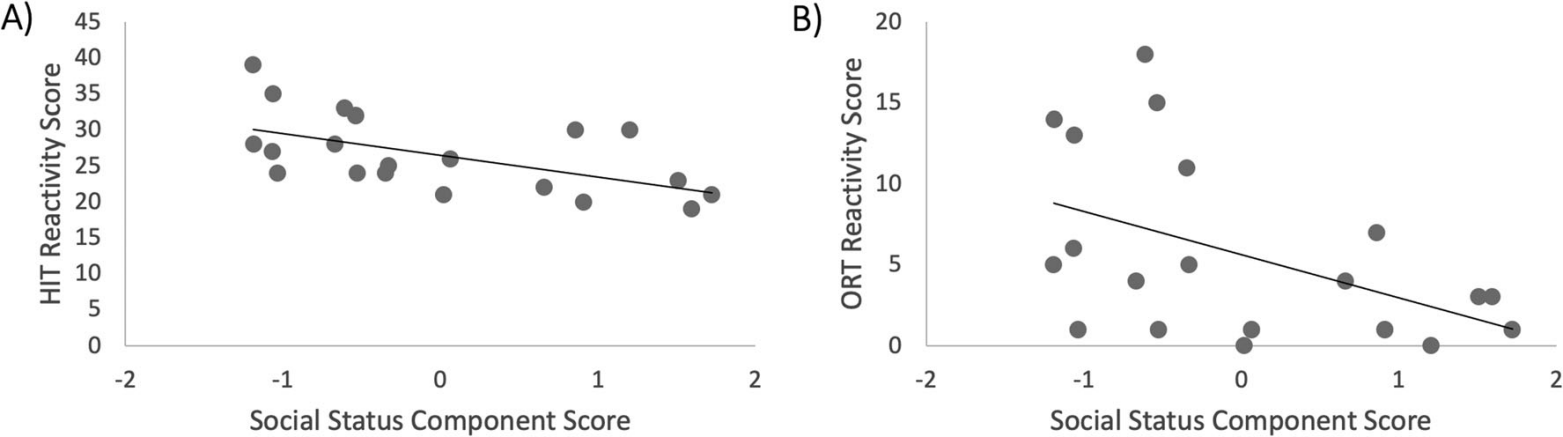
The relationship between social status and affective reactivity on the HIT (**a**) and ORT (**b**). Individual animals’ regression scores from component 1 of the principal component analysis are plotted on the *x*-axis

We regressed the two component scores from the principal components analysis and dominance probability onto the total affective reactivity (summed across conditions) for HIT and ORT using stepwise regression (inclusion criterion set at *p* <0.05). Component 1 (hereafter *social status*) was a significant predictor of both HIT and ORT reactivity, such that greater social status predicted lower reactivity. HIT: *F*(1, 19) = 8.43, *p*=0.009; *β* = −0.57, *t*=−2.90, *p*=0.009. ORT: *F*(1, 19) = 5.40, *p*=0.032; *β* = −0.48, *t*=−2.32, *p*=0.032. Neither component 2 (social motivation) nor dominance probability were significant predictors of either HIT or ORT reactivity and so were not maintained in the model. For HIT, social motivation: *β* = 0.074, *t*=0.37, *p*=0.72; dominance probability: *β* = 0.045, *t*=0.27, *p*=0.83. For ORT, social motivation: *β* = 0.13, *t*=0.62, *p*=0.54; dominance probability: *β* = 0.032, *t*=0.15, *p*=0.89 (Fig. 2).

## Discussion

Our data demonstrate that affective reactivity is related to social status in rhesus monkeys. Affective reactivity was indexed using two classic affective reactivity tests, human intruder and object responsiveness. Reactivity during those tasks is thought to be related to the neurobiology that subserves affect, specifically amygdala structure and function (Bliss-Moreau et al., 2011; Bliss-Moreau et al., 2010; Kalin et al., 2001; Kalin et al., 2004; Mason et al., 2006; Medina et al., 2021). Reactivity is also predictive of a number of behavioral and physiological outcomes, including cooperative training outcomes (Bliss-Moreau & Moadab, 2016) and cortisol reactivity (Hamel et al., 2017), and is predicted by an animals’ early social experiences. Infants who are raised in restricted social environments are more reactive than peers raised in enriched social environments (Gottlieb & Capitanio, 2013b).

In the present experiment, the social status component reflected rank, age, primary and secondary incoming (or recieved) prosocial bonds, the anthropomorphized equivalent of animals who perceive the subjects as “friends” and the “friends of those friends,” and the centrality or importance of their social role. These are measures that are not easily obtained in humans, equivalent to asking “how many people consider you their friend?” and “how many friends do those people have?” In contrast, the social motivation measure that is more easily quantified in humans (i.e., “how many friends do you have?”) loaded onto the second component along with the number of “friends of friends.” Social motivation was not predictive of reactivity. Both the social status and social motivation components were multi-faceted (included more than one index) which ultimately means that we are unable to determine what particular feature of social status (betweenness centrality? number of monkeys who groom the subject? age?) was most predictive of reactivity. In our view, this reflects the reality that the nature of an individual’s social life is multiply determined — both for monkeys and for humans. That said, future work in groups where these features do not covary, either as a natural product of the group structure or because we have experimentally manipulated group structure with regards to age, might allow us to tease apart the impact of the different components on reactivity. Similarly, certainty of status did not predict of reactivity, but it is possible that it might in other groups where there is a greater range or different distribution of dominance probability across individuals.

These findings speak to understanding individual differences in affective reactivity which have a wide variety of consequences for health. For example, people who experience more robust negative affective responses to stress were more likely to report having an affective disorder (Charles et al., 2013) or a chronic health condition (Piazza et al., 2013) ten years later. Similarly, in a large-scale study of aging adults, mortality risk is increased for people with illness as a function of the magnitude of their affective responses to stressful situations (Chiang et al., 2017). What our findings suggest is that aspects of social environment and an individual’s social behavior are related to the magnitude of his affective response to threat. What is unclear from these data is the directionality of the effects and whether these effects are affect general or specific to negative valence. Such directionality could be determined in studies that create new groups with animals who have varied affective reactivity or animals who have similar affective reactivity but different social status in their original groups. It is worth noting that our affective reactivity index here reflects two point estimates, leaving open questions about how prolonged negative affective reactivity over time (e.g., stress) might relate to social roles and social status as we define it here. A good deal of literature exists documenting relationships between rank and stress (e.g., lower ranking animals may have higher levels of glucocorticoids; see Sapolsky, 2004 for a review; although see Cohen et al., 2019 for a discussion of how little consensus there is about what “stress” is).

Our decision to use information about grooming relationships to quantify the number of primary and secondary social bonds and the importance of the animals’ social role was predicated on translatability to human studies. Indeed, data on aggression and antagonism were also collected as part of the larger social network study of this group (Balasubramaniam et al., 2016; Vandeleest et al., 2016, 2020) but we were eager to compute metrics that we might also be able to evaluate from self-reports made by human participants, allowing us to carry out simultaneous studies across species. Grooming in nonhuman primates is one of the most important behaviors animals use to generate, sustain, and heal social bonds. Nonhuman primates spend around 20% of their days engaged in social grooming (Henzi & Barrett, 1999) and time spent grooming is positively correlated with average group size, suggesting grooming has a social function (Dunbar, 1991). Evidence that monkeys often groom up the hierarchy (Schino, 2001) as a means by which to establish social support from higher ranking animals (Schino, 2007) is consistent with our finding that rank and the number of primary and secondary groomers a subject had were correlated. Directionality of grooming also has physiological consequences that may speak to our affective reactivity effects. For example, being groomed, but not grooming others, has been demonstrated to reduce animals’ heart rates (Boccia et al., 1989). Regardless of differential patterns and functions of grooming, grooming between two individuals has been interpreted as evidence of rhesus monkeys’ “friendship” (Ellis et al., 2019; Weinstein & Capitanio, 2012).

The vast majority of studies evaluating psychological and physiological functions as related to social network parameters in people ask participants to self-report on the number of friends, acquaintances, or contacts they have (e.g., using a tool like the Social Network Index: Bickart et al., 2014; Cohen et al., 1997) or self-report on or extract the number of social media contacts they have (e.g., Kanai et al., 2012; Lewis et al., 2008, but see Morelli et al., 2017 for an alternative approach). Given the findings in the human literature, we expected that we might see a relationship between affective reactivity and out-degree (“outgoing”) grooming partners as this is likely the closest proxy for self-reported close social contacts in humans. No such relationship was observed, and rather affective reactivity was predicted by the component that included in-degree (“incoming”) grooming partners, a feature of social networks not often captured in studies of people. Our data emphasize the importance of being able to model closed social systems in animal models and also the importance of studies in humans that can capture unreciprocated and indirect relationships, such as those that evaluate social networks in college dormitories and via social media accounts.

The limitations of this study point to clear future directions. Our sample size of 20 adult male monkeys is consistent with the sample size of many nonhuman primate studies but is much smaller than is ideal for studies of individual differences. The sample size was determined by including all adult male monkeys in the cage who were available for study and thus, was not selective. Future studies should both increase the sample size and include female animals, who are born into their rank rather than earning it, and who rarely leave their natal group (Cawthon Lang, 2005; Fooden, 2000). Thus, females may evidence different relationships between their social network status and psychological function than do males. Future work should also increase the group-level sample size. Different social groups of rhesus monkeys have inherently different social network structures that include variation in terms of what type of animals have the most central roles, how clear and stable ordinal rank is, variation in dominance probability, variation in how aggression is controlled or “policed,” and how roles vary over time, etc. (Balasubramaniam et al., 2018; Beisner et al., 2015; Beisner et al., 2020; Beisner & McCowan, 2013; Brent et al., 2013; Vandeleest et al., 2016). This variation could be driven by variation in animals’ psychological function, could drive variation in animals’ psychological function, or some combination of the two. Finally, we elected to focus on the affiliative network parameters in this report because they most closely mirror the sorts of social network measures that are collected in humans — but future studies should investigate networks related to negative interactions as well.

Another limitation of the present study is that animals were transported from their large group home to the laboratory for affective reactivity testing. Animals at our center experience relocations like this from time-to-time, but a relocation nonetheless has the potential to be stressful and there may be individual differences in that experience. To guard against this, we brought animals into the lab in pairs, specifically with another male with whom they affiliated, based on data from our center demonstrating that access to a social partner buffers the impact of relocation from outdoor groups to indoors (Hennessy et al., 2017). If this was the case, then our index of affective reactivity must be considered in context. Nevertheless, the affective reactivity pattern observed in HIT was consistent with what we have observed in previous studies: animals were most reactive in the Stare Near condition, and least reactive in both Profile conditions (Bliss-Moreau & Baxter, 2018). All monkeys evidenced this pattern, suggesting that the impact of the relocation to the laboratory was consistent across animals. We did not observe differential reactivity in the ORT, perhaps because the ostensibly threatening stimuli were not potent enough (and thus treated like any other novel object) or perhaps because the task occurred on day 2 of testing. We elected to include it because there were individual differences in ORT and they were predicted by the same variables that predicted variation in HIT. Methods for carrying out affective reactivity testing in animals’ home environments would allow for in situ evaluations and also to determine the extent to which variation in affective reactivity is contextually dependent.

Finally, it is important to note that while rhesus monkeys are arguably a better model for human neurobiology and affective and social processing than are rodents, the structure of their social societies does differ from humans in important ways which could impact the translatability of our results. Rhesus monkeys live in matrilineal societies, where females remain in their natal groups and males migrate from the troop at sexual maturity (for a discussion Suomi, 2005; Cawthon Lang, 2005; Fooden, 2000; Melnick et al., 1984). As a result, females’ ranks are determined by their mother’s rank and their birth timing (relative to female siblings, newest born female child gets the rank [mother’s rank — 1]) while males must navigate the social dynamics of their new groups in order to earn their ranks (Cawthon Lang, 2005; Manson, 1998; Melnick et al., 1984). It was with these social features in mind that we elected to focus the current investigation on male animals, because rank is not a birth right for males and thus variation in social status relative to behavioral outcomes could not be explained solely by matrilineal influences. Because of these differences between males and females, however, it is possible that the social status component may differ across males and female (e.g., we would not expect rank and age to be correlated since very young females born to high-ranking females also have high ranks) and also possible that there could be sex differences in the impact of social status on affective reactivity. This variation may be species specific given the specific social structure of rhesus monkeys. Replicating this experiment in a sample that includes females in order should help clarify these issues and carrying out the work with humans that mirrors the current design should clarify the translatability between species.

In closing, our results suggest that animals with greater social status had lower affective reactivity during a series of laboratory-based tasks that required being temporarily removed from their home environment. Our social status component included age — a feature of individual animals that was independent of the social group — as well as ordinal rank, the number of grooming partners from which grooming was received, secondary social reach and betweenness, an index of how central or important the individuals’ social role is in the network — all of which are dependent on the structure of the social group. Future work will investigate the causal directionality of these relationships in groups that vary in social properties in order to understand how an individual’s affective reactivity emerges in social context and influences his or her social role.

## Additional Information

### Data Availability

Data reported in this paper are available here https://osf.io/7t6jf/

### Code Availability

n/a
